# Comparison of Safety and Effectiveness of Local or General Anesthesia after Transcatheter Aortic Valve Implantation: A Systematic Review and Meta-Analysis

**DOI:** 10.3390/jcm12020508

**Published:** 2023-01-07

**Authors:** Luchen Wang, Yanxiang Liu, Haoyu Gao, Bowen Zhang, Sangyu Zhou, Mingxin Xie, Xiaogang Sun

**Affiliations:** Aortic and Vascular Surgery Center, Fuwai Hospital, National Center for Cardiovascular Diseases, Chinese Academy of Medical Sciences and Peking Union Medical College, Beijing 100037, China

**Keywords:** transcatheter aortic valve implantation, general anesthesia, local anesthesia, conscious sedation, meta-analysis

## Abstract

It remains controversial to choose anesthesia for transcatheter aortic valve implantation (TAVI). A meta-analysis of cohort studies was conducted to assess the efficacy and safety of local anesthesia (LA) compared to general anesthesia (GA) in TAVI. All relevant studies published from 1 January 2002, to 31 June 2022, were searched in Ovid, PubMed, Embase, Web of Science, and Cochrane Library. A total of 34 studies involving 23,480 patients were included in the meta-analysis. TAVI with LA was associated with a significant reduction in hospital stay [WMD = −2.48, 95% CI (−2.80, −2.16), *p* < 0.00001], operative [WMD = −12.25, 95% CI (−13.73, −10.78), *p* < 0.00001] and fluoroscopy time [WMD = −3.30, 95% CI (−5.40, −1.19), *p* = 0.002], and an increased risk of acute kidney injury [OR = 1.31, 95% CI (1.01, 1.69), *p* = 0.04] and a reduced incidence of major bleeding [OR = 0.59, 95% CI (0.46, 0.75), *p* < 0.0001] and the use of cardiovascular drugs [OR = 0.17, 95% CI (0.05, 0.57), *p* = 0.004]. No differences were found between LA and GA for 30-day mortality, procedural success rate, myocardial infarction, permanent pacemaker implantation, paravalvular leak, shock, and cerebrovascular events. Overall, 4.4% of LA converted to GA. Based on current evidence, our results suggested that LA strategies reduced hospital stay, operative time, fluoroscopy time, cardiovascular drug consumption, and major bleeding rates in patients undergoing TAVI but led to increased acute kidney injury rates. Further studies and randomized trials are required to verify the presented findings and to identify patients who might benefit from LA.

## 1. Introduction

Aortic stenosis (AS) is the most common valvular heart disease in the Western world, with a prevalence of 2% among patients over 65 and 4% among patients over 85 years of age [1,2]. About 50% of AS patients with symptoms die within two years [3,4]. As a result, aortic valve replacement (AVR) is recommended as a Class I indication for the treatment of AS patients with symptoms [5,6]. The presence of severe comorbidities, advanced age, and/or left ventricular dysfunction prevent more than 30% of patients with severe symptoms of AS from undergoing surgical aortic valve replacement (SAVR) [7]. Transcatheter aortic valve implantation (TAVI) has become an effective therapy for patients with severe symptomatic AS due to its quick operation time and low invasiveness. Furthermore, the 2020 AHA/ACC guidelines recommend TAVI as the primary intervention, as well as an alternative operation to SAVR in patients with high operative risk factors and intermediate operative risk [8].

In 2002, TAVI was first performed under conscious sedation (CS) [9]. Traditionally, excessive depth of anesthesia has been associated with higher mortality and delirium from an anesthetic point of view [10]. With the improvement in the level of procedures, some surgeons have gradually begun to experiment with non-general anesthesia methods. Successful cases of local anesthesia (LA) with CS for TAVI have been reported in both Europe and the United States [11], which can avoid the hemodynamic instability [12] and pulmonary complications [13] related to general anesthesia (GA) and concomitant positive pressure ventilation (PPV). However, GA in TAVI also has advantages, such as easy access to perioperative transesophageal echocardiography and easier management of surgical complications [14]. Controversy remains regarding the impact of the type of anesthesia used in TAVI on surgical safety and clinical outcomes. Thus, we evaluated whether the use of LA has a favorable impact on complications and prognosis in patients with severe AS when compared to TAVI under GA.

## 2. Methods

The reporting of this systematic review and meta-analysis follows the Preferred Reporting Items for Systematic Reviews and Meta-Analyses (PRISMA) and Assessing Methodological Quality of Systematic Reviews (AMSTAR) guidelines [15]. This project has been registered with PROSPERO (CRD42021221777).

### 2.1. Data Sources and Search Strategy

The literature search was performed through the Ovid, PubMed, Embase, ClinicalTrials.gov, Cochrane Library, and Web of Science databases to collect the relevant studies published from 1 January 2002 to 31 June 2022. Moreover, citations from articles were retrieved to identify relevant studies that were not included in the initial literature search. Details about the search algorithm can be found in the Supplementary Materials in Word format. All included articles had no language or sample-size restrictions.

### 2.2. Inclusion and Exclusion Criteria

Selected papers must include the following information to satisfy the inclusion criteria: comparison data of TAVI procedure that a group of patients used LA with or without CS (±CS) approach, while another group received GA; meanwhile, the primary outcome parameters were also reported. Articles with no direct comparison data of LA (±CS) and GA in the studies, patients younger than 18 years old and letters, case reports, reviews, comments, and meeting abstracts were excluded from this study.

### 2.3. Data Extraction and Quality Evaluation

An independent process was performed by two authors (LW and HG) to extract relevant information from the articles using a prepared standardized extraction database, including the baseline clinical characteristics and outcome measures of the study population. The disagreement was resolved by the senior author (XS). We contacted the original author via e-mail to request the lack of essential data in some papers. Studies were not blinded to the author, journal, or institution.

### 2.4. Outcomes and Definitions

The primary outcome measures were hospital length of stay, operation time, 30-day mortality, use of cardiovascular drugs, the incidence of paravalvular leakage (PVL), the rate of implantation of permanent pacemaker (PPM), fluoroscopy time, stroke rate, incidence of myocardial infarction (MI), acute kidney injury (AKI), major bleeding (MB), conversion from LA to GA, and procedural success.

### 2.5. Risk of Bias Assessment

Individual studies were assessed in duplicate using the Cochrane Collaboration’s risk of bias tool [16]. The following aspects were evaluated for each survey: random sequence generation, allocation concealment, blinding of participants and personnel, blinding of outcome assessment, incomplete outcome data, and selective outcome reporting. Depending upon the level of bias judgement, low bias judgment, unclear bias judgement (indicating unclear or unknown risks of bias), and high bias judgement may be present.

### 2.6. Statistical Analysis

All data were analyzed by Review Manager (RevMan) version 5.4 (The Cochrane Collaboration, Copenhagen, Denmark) and Stata SE 16.0 (Stata Corporation, College Station, TX, USA). The odds ratio (OR) with 95% confidence intervals (CI) were estimated for dichotomous data and standard mean difference (SMD) with 95% CI for continuous data, respectively. Study heterogeneity was tested by a formal Q statistical test and I^2^ statistical test (test level = 0.1). A *p*-value > 0.1 and I^2^ < 50% showed that the included studies were homogeneous [17]. Meta-analysis was performed using the Mantel–Haenszel fixed-effects model. If there was heterogeneity between the studies, after excluding the influence of apparent causes of heterogeneity, the Der Simonian and Laird random-effects model was used for meta-analysis. Subgroup and meta-regression analyses were conducted to explore the possible source of heterogeneity. Risk of publication bias for studies will be assessed using funnel plots, and Egger’s test was employed to examine the publication bias when there were at least 10 studies. A significance level of α = 0.05 was set for all analyses. Sensitivity analysis was used to assess whether the results were robust and to assess sources of heterogeneity. 

## 3. Results

### 3.1. Search Results

**Figure 1:**
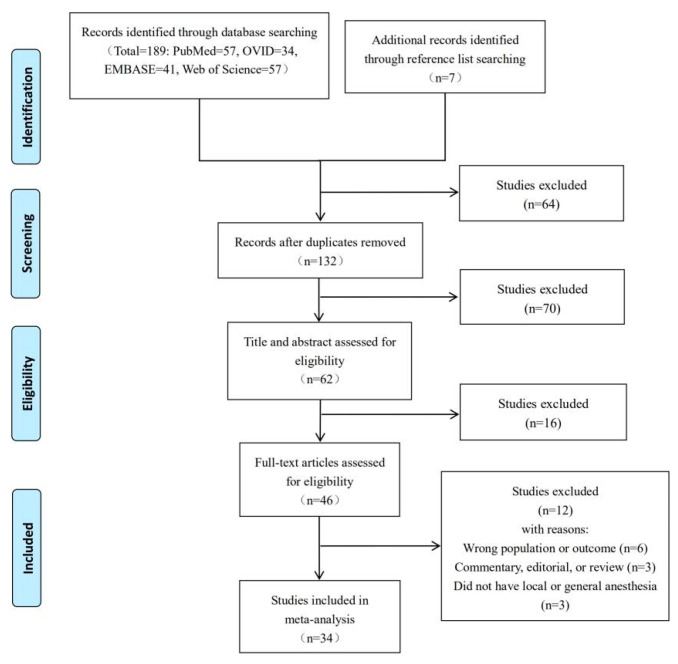
Flow diagram of study selection process corresponding to PRISMA statement.

A total of 196 relevant studies were retrieved, followed by a sequential process of removing duplicates, screening titles and abstracts, and finally screening full-text studies to eliminate all irrelevant articles. For the final analysis, 34 studies were included, including 3 randomized controlled trials, 4 prospective cohort studies, 24 retrospective cohort studies, and 3 case-control studies involving 23,480 patients (Figure 1) [18,19,20,21,22,23,24,25,26,27,28,29,30,31,32,33,34,35,36,37,38,39,40,41,42,43,44,45,46,47,48,49,50,51].

### 3.2. Study Characteristics

**Figure 2:**
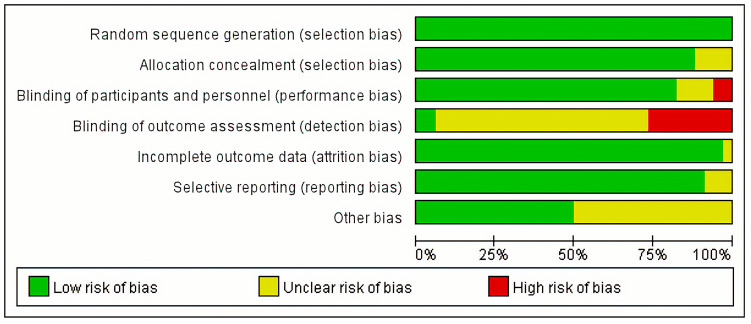
Risk of bias graph: review authors’ judgements about each risk of bias item presented as percentages across all included studies.

The basic characteristics of the included studies, which were published between 2010 and 2020, are summarized in Table 1 [18,19,20,21,22,23,24,25,26,27,28,29,30,31,32,33,34,35,36,37,38,39,40,41,42,43,44,45,46,47,48,49,50,51]. The sample sizes of the individual experiments ranged from 49 to 5248. Basic informations about the patient population enrolled in the studies are presented in Table 2 [18,19,20,21,22,23,24,25,26,27,28,29,30,31,32,33,34,35,36,37,38,39,40,41,42,43,44,45,46,47,48,49,50,51]. A total of 21,108 patients were involved, with an overall mean age of 81.6 years. The TAVI procedure was performed on all patients. Figure 2 and Supplementary Figure S1 show the bias risk for all included studies. Most of the bias risks relate to blinding of outcome assessment (detection bias), with nearly 26% of high risks and nearly 68% of unclear risks. The analysis of selection bias showed no high risk of bias, with a few unclear risks. Overall, less than 10% of the risk of bias was present in the remaining fields of bias, including performance, attrition, reporting, and other bias.

### 3.3. Length of Stay

**Figure 3:**
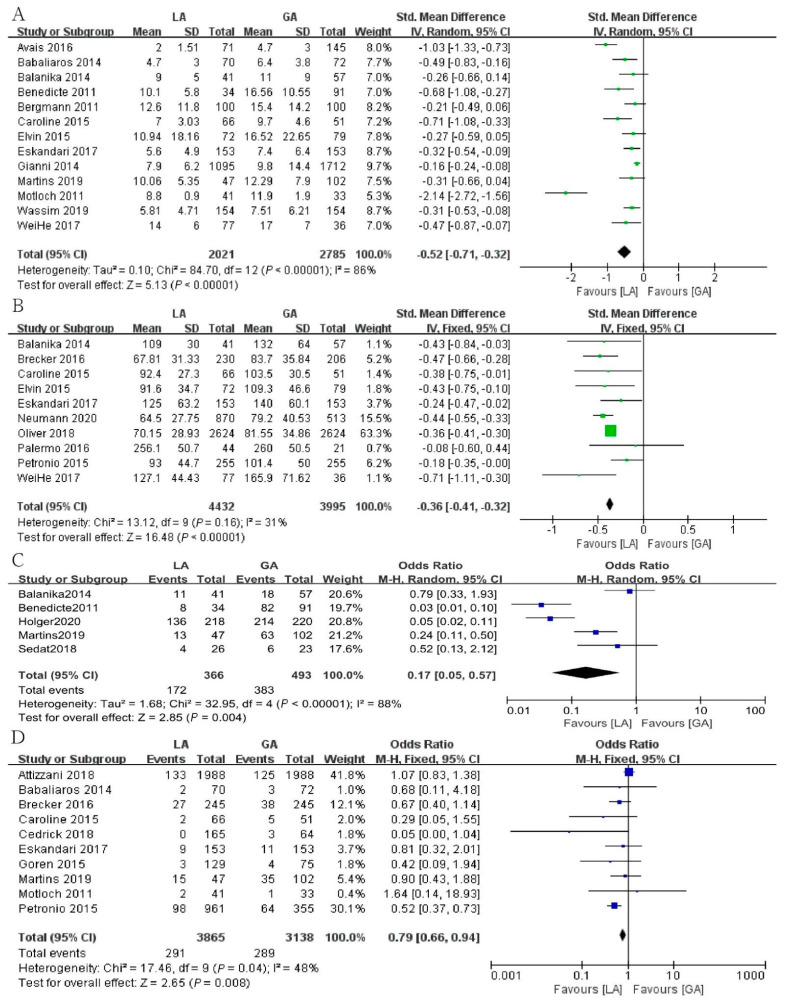
Forest plot of (**A**) length of stay, (**B**) the procedural time, (**C**) use of cardiovascular drugs, (**D**) the incidence of major bleeding. Green symbols: continuous data; blue symbols: dichotomous data; black symbols: other type data.

A total of 13 studies reported length of stay, involving a total of 4806 patients. LA group has been associated with a significantly shorter hospital stay compared with GA group [SMD = −0.52, 95% CI (−0.71, −0.32), *p* < 0.00001, I^2^ = 86%] (Figure 3A). 

### 3.4. Procedural Time

Including a total of 8427 patients, 10 studies analyzed procedural time. For TAVI surgery, GA was associated with a significantly longer procedure time when compared to LA [SMD = −0.36, 95% CI (−0.41, −0.32), *p* < 0.00001, I^2^ = 31%] (Figure 3B).

### 3.5. Using of Cardiovascular Drugs

Five studies reported data on the use of inotropes and/or vasoactive medication. LA group was lower in the rate of using cardiovascular drugs, and the difference was statistically significant [OR = 0.17, 95% CI (0.05, 0.57), *p* = 0.004, I^2^ = 88%] (Figure 3C). 

### 3.6. Incidence of MB

A total of 10 studies reported the incidence of MB, involving a total of 7003 patients. The incidence of MB in LA group was lower than GA group, and the difference was statistically significant [OR = 0.79, 95% CI (0.66, 0.94), *p* = 0.008, I^2^ = 48%] (Figure 3D).

### 3.7. 30-Day Mortality

**Figure 4:**
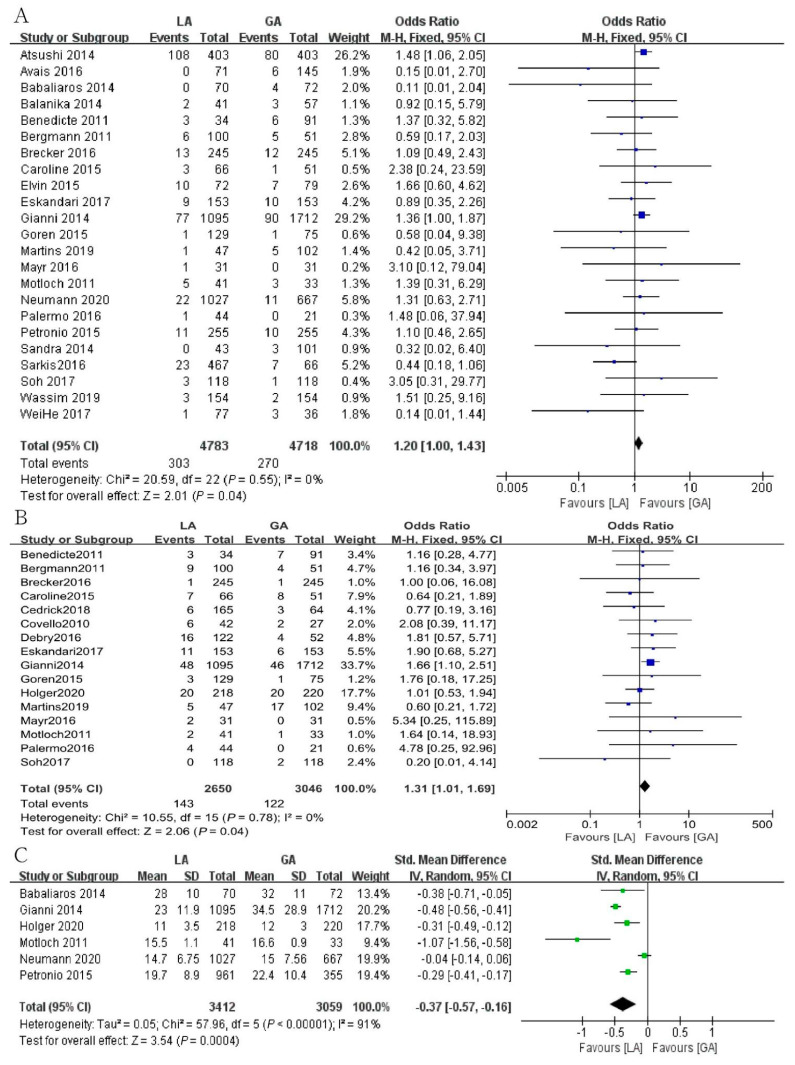
Forest plot of (**A**) 30-day mortality. (**B**) The incidence of acute kidney injury. (**C**) The fluoroscopy time. Green symbols: continuous data; blue symbols: dichotomous data; black symbols: other type data.

There were no remarkable differences regarding 30-day mortality rate between LA (±CS) group and GA group. A total of 303 of 4783 patients (6.33%) in the LA group and 270 of 4718 patients (5.72%) in the GA group were reported to be dead [OR = 1.20, 95% CI (1.00, 1.43), *p* = 0.04, I^2^ = 0] (Figure 4A).

### 3.8. Incidence of AKI

The incidence of AKI in a total of 5696 patients in 16 studies was analyzed. In the LA group, the incidence was 143 of 2650 (5.4%), while in the GA group, it was 122 of 3046 (4.0%). There were significant differences regarding the incidence of AKI between these two groups [OR = 1.31, 95% CI (1.01, 1.69), *p* = 0.04, I^2^ = 0] (Figure 4B).

### 3.9. Fluoroscopy Time

A total of 6471 patients were included, with six studies analyzing fluoroscopy time. Compared to the GA group, fluoroscopy time was significantly reduced in the LA group [SMD = −0.37, 95% CI (−0.57, −0.16), *p* = 0.0004, I^2^ = 91%] (Figure 4C).

### 3.10. Other Outcomes

**Figure 5:**
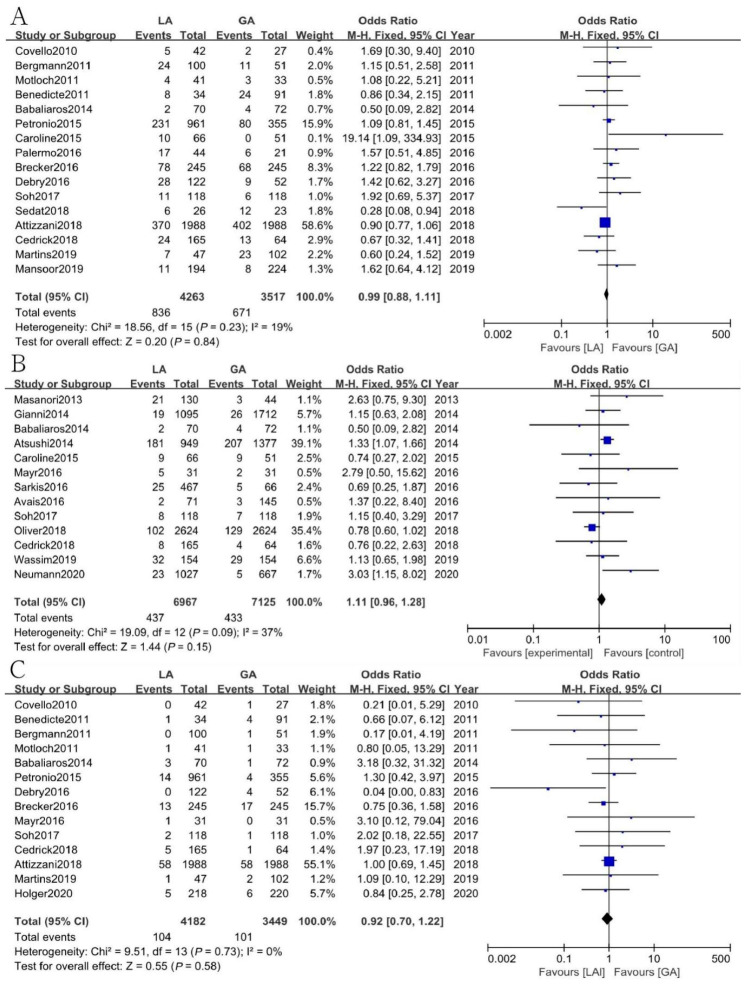
Forest plot of (**A**) permanent pacemaker implantation rate. (**B**) Paravalvular leakage. (**C**) Shock. Green symbols: continuous data; blue symbols: dichotomous data; black symbols: other type data.

**Figure 6:**
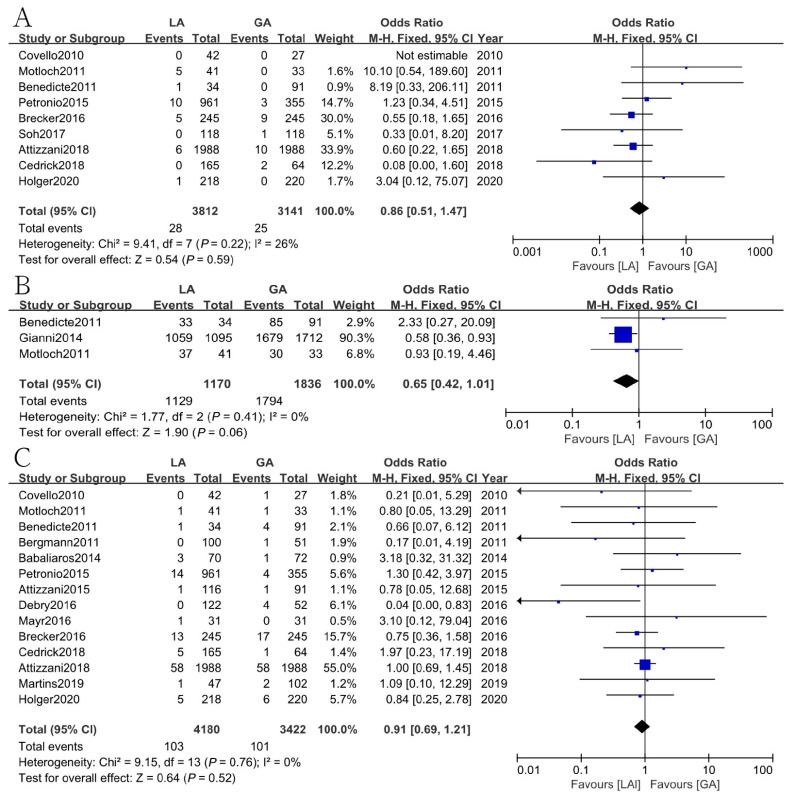
Forest plots of (**A**) myocardial infarction. (**B**) The rate of procedural success. (**C**) The rate of cerebrovascular events. Green symbols: continuous data; blue symbols: dichotomous data; black symbols: other type data.

The results showed that a total of 182 out of 4157 patients (4.4%, CI: 3.8–5.1) required a change in anesthesia strategy from LA to GA. The most common reasons for changing the anesthetic management were restlessness, hemodynamic compromise, and procedural complications (Supplementary Tables S1 and S2). There were also some other outcomes analyzed to compare the safety of local anesthesia (LA) with or without conscious sedation (CS) and general anesthesia (GA) for the TAVI procedure, but no significant differences were found regarding PPM [OR = 0.99, 95% CI (0.88, 1.11), *p* = 0.84, I^2^ = 19%], PVL [OR = 1.11, 95% CI (0.96, 1.28), *p* = 0.15, I^2^ = 37%], shock [OR = 0.92, 95% CI (0.70, 1.22), *p* = 0.58, I^2^ = 0], MI [OR = 0.86, 95% CI (0.51, 1.47), *p* = 0.59, I^2^ = 26%], the rate of procedural success [OR = 0.65, 95% CI (0.42, 1.01), *p* = 0.06, I^2^ = 0], or the rate of cerebrovascular events [OR = 0.91, 95% CI(0.69, 1.21), *p* = 0.52, I^2^ = 0] between the two groups (Figure 5 and Figure 6).

### 3.11. Subgroup Analysis

**Figure 7:**
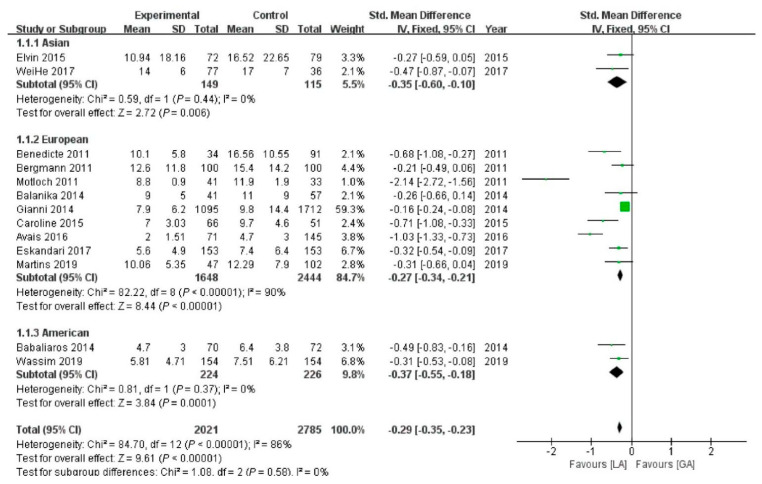
Subgroup analysis of race in length of stay. Green symbols: continuous data; blue symbols: dichotomous data; black symbols: other type data.

We speculated that race might be sources of heterogeneity and performed subgroup analyses. Race had influence on the length of stay. For Asian people, SMD is −0.35 days; in Europeans, SMD is −0.27 days, while in Americans, SMD is −0.37 days. These differences were consistently statistically significant (Figure 7). 

### 3.12. Meta-Regression for the Potential Sources of Heterogeneity

In the random-effect univariate meta-regression analysis of cardiovascular drug use, the rates of PVL and fluoroscopy time, age, EuroScore, DM, and LVEF were considered separately. Nevertheless, the results were not statistically significant.

### 3.13. Publication Bias Assessment and Sensitivity Analysis

The funnel plot of the length of stay, procedural time, the incidence of AKI, and the incidence of MB have no obvious asymmetry. Results also showed that there was no apparent publication bias in the length of stay (Begg’s *p* = 0.161), procedural time (Begg’s *p* = 0.592), the incidence of AKI (Begg’s *p* = 0.620), the incidence of MB (Begg’s *p* = 0.592), the incidence of PPM (Begg’s *p* = 0.620), the incidence of shock (Begg’s *p* = 0.584), the rate of cerebrovascular enents (Begg’s *p* = 0.511), or the rate of PVL (Begg’s *p* = 1). For other outcomes, due to the small number of included studies, no Begger test or Egger test was performed, but there was no obvious asymmetry in the funnel plot. After excluding the studies of Attizzani GF et al. [45], I^2^ = 0%, the incidence of MB in the LA group was significantly lower than that in the GA group (*p* < 0.0001). This is consistent with the results of the sensitivity analysis. 

## 4. Discussion

Historically, GA was the standard anesthetic strategy for TAVI surgery, but as surgical instruments have improved and as medical teams have become more experienced, LA has become an increasingly popular anesthetic technique for TAVI surgery as an alternative to GA [26]. With GA, transesophageal echocardiography (TEE) can be performed in real time, allowing for accurate assessment of valve conditions, as well as early detection of complications, such as aortic dissection, thrombosis, and valve embolisms. Moreover, GA prevents patient movement, which may interfere with valve deployment under rapid pacing. Furthermore, GA allows for rapid conversion to bailout in case of perioperative complications [52]. Nevertheless, GA is associated with a relatively high incidence of perioperative mortality and delirium due to excessive depth of anesthesia [53]. In contrast, LA maintains intraoperative hemodynamic stability better, uses less PPV, and reduces the length of hospital stay while shortening surgery time and accelerating patient recovery [54]. Therefore, to compare the safety and efficacy of GA and LA in patients with severe AS undergoing TAVI, we performed an updated meta-analysis by comprehensively and systematically evaluating the existing relevant studies. Based on our findings, LA strategy was associated with a significant reduction in length of stay, operative time, fluoroscopy time, cardiovascular drug use, and MB incidence during TAVI. The LA group also had a relatively low incidence of MI, shock, and cerebrovascular events, as well as a relatively high incidence of AKI and PVL, but none of these differences were statistically significant.

Previous research has shown that reducing hospital stay and procedure time is often accompanied by decreased postoperative complications, such as hospital-acquired infections and quicker recovery speeds for patients [55]. As a consequence, we compared the prognosis of patients undergoing TAVI between LA and GA based on the length of hospital stay and the procedural time. According to Neumann et al. [49], the CS group had a shorter procedure time, used 10 mL less contrast media, and suffered a lower rate of post-dilatation than the GA group. Petronio et al. [28] found a significantly longer procedural time in the GA group. Our results showed that the LA resulted in shorter length of stay in hospital and procedural time in patients compared with the GA. These results may relate to less hemodynamic instability and a lower requirement for vasoactive medications associated with GA, with an overall lower patient acuity level. Of course, the most probable reason for lower procedural time is that GA needs anesthesia induction and recovery time. Additionally, a shorter procedural time and hospital stay always means less treatment costs, which is beneficial for patients.

A number of studies [56,57,58] have found no significant difference in 30-day mortality between the above two anesthetic strategies for TAVI patients. A previous meta-analysis [59] pointed out that the anesthesia method did not influence the average 30-day mortality rate, which was 100/1532 patients in the LA group and 114/2061 patients in the GA-group [RR = 0.91, 95% CI (0.53, 1.56), *p* = 0.72]. The SOURCE 3 Registry [60] showed that the CS group had 2.1% all-cause mortality within 30 days, while the GS group had 1.7% (*p* = 0.47). However, a different outcome was reached in a randomized controlled trial conducted by Thiele H et al. [50], who found that all-cause mortality was significantly higher in the LA group of patients undergoing TAVI than in the GA group [3.2% vs. 2.3%, *p* < 0.001]. In our results, no remarkable differences regarding 30-day mortality rate between LA (±CS) group and GA group were found. Perhaps there is no association, so we need larger multicenter studies to verify the relationship.

The TAVI procedure involves several short dangerous episodes, including extreme hypotension and global ischemia (rapid ventricular pacing, balloon valvuloplasty, valve implantation), coronary embolism, and some degree of myocardial tissue compression (balloon and valve prosthesis), which are all potential causes of myocardial injury. Patients also can die from multiorgan failure, major stroke, pulmonary embolism, heart failure, left ventricle perforation, or cardiogenic shock [61]. Thus, we further evaluated the effect of LA and GA anesthesia strategies on TAVI-related complications, and the results indicated that the LA group had lower rates of MI [OR = 0.86, 95% CI (0.51, 1.47), *p* = 0.59], shock [OR = 0.92, 95% CI (0.70, 1.22), *p* = 0.58] and cerebrovascular events [OR = 0.91, 95% CI (0.69, 1.21), *p* = 0.52] than the GA group, but the differences were not statistically significant. 

Patients undergoing TAVI face a non-negligible risk of postoperative bleeding and transfusion, with the incidence of MB ranging from 15% to 20% [62]. Our results showed that the incidence of MB was significantly lower in the LA group of patients undergoing TAVI than in GA.

Regardless of choosing the LA or GA as anesthesia method, there were no remarkable differences regarding the procedural success rate [OR = 0.65, 95% CI (0.42, 1.01), *p* = 0.06]. During TAVI procedure, hemodynamic instability and hypotension deserve mentioning. The main intraoperative risk during TAVI is hemodynamic instability, and it is crucial for anesthesiologists and cardiologists to promptly diagnose the causes of hemodynamic instability that may need rapid intervention, using invasive arterial monitoring, TEE, TTE, fluoroscopy, and contrast angiography. Additionally, being careful about hypotension due to rapid ventricular pacing during balloon aortic valvuloplasty is essential. 

Although there was no significant difference in procedural success, our results found that the rate of using inotropes and/or vasoactive medication was significantly lower in the LA group [OR = 0.17, 95% CI (0.05, 0.57), *p* = 0.004], which may suggest a simpler and smoother procedure with LA. 

A multifactorial basis for renal injury after TAVI surgery includes nephrotoxic contrast media, diffuse atherosclerosis, and intraoperative hemorrhage. Additionally, debris embolization during valvuloplasty and valve deployment, anesthetic-induced hypotensive episodes, rapid right ventricular pacing, and pre-existing conditions such as diabetes, hypertension, and renal impairment can all increase postoperative AKI risk. In our results, the incidence of AKI in the LA group was higher than that of the GA group [OR = 1.31, 95% CI (1.01, 1.69), *p* = 0.04]. As a result of an RCT study that included 2807 TAVI patients undergoing various anesthetic strategies, Dall’Ara et al. [26] reported that AKI was more likely to be diagnosed in the LA group, while transient or permanent renal replacement therapy was required more often in the GA group. However, due to the earlier publication of this study and the larger number of patients in the GA group, the results are suspect due to this bias. Thiele et al. [50] reported in an RCT that the incidence of AKI at different stages was similar between the LA and GA groups, with stage 1, 2, and 3 AKI occurring at 4.7% and 5.5%, 1.9% and 1.8%, and 2.4% and 1.8%, respectively. Contrast-induced nephropathy after TAVI is controversial, and it is still unknown whether this nephropathy is associative or causative. Zaouter et al. [44] reported they did not reveal disparities in the incidence of AKI Stage 3 and the requirement for RCT between the two groups with different anesthetic strategies.

The occurrence of atrioventricular block (AVB) after TAVI is a relatively common problem and is one of the most concerning issues for cardiologists. High-grade AVB and new-onset left bundle branch block (LBBB) remain the most frequent indications for PPM. The rate of PPM can reflect the occurrence of AVB after TAVI to some extent, thus we use it as an outcome to compare the safety between LA and GA. Palermo et al. [35] analyzed both patients who received Medtronic Core Valve and found a high rate of PPM insertion in the LA group, but it did not achieve statistical significance. Our results also indicated no statistical difference in the incidence of PPM between the two groups. However, this conclusion should be taken rigorously because the incidence of AVB is different between self-expandable valves and balloon-expandable valves, as well as being different in other generations [63]. After TAVI, pre-procedural predictors of new-onset LBBB include female sex, diabetes mellitus, prior CABG, first degree AVB, prolonged QRS duration, aortic annulus calcification, and larger left ventricular end-diastolic volume. Procedural associated factors including Core Valve implantation, transapical access, pre-dilation, oversizing, and lower implantation depth [64].

In recent years, there have been cases of conversion from LA to GA during TAVI. A major benefit of applying GA during TAVI is improved management of sudden and life-threatening complications. Based on this meta-analysis, we discovered that conversion from LA to GA is infrequent and primarily related to restlessness, hemodynamic compromise, and procedural complications. Moreover, no data exist on the increased risk of death associated with conversion from LA to GA, indicating that conversion seems safe in the hands of a prepared anesthesiologist. For this reason, we recommend that experienced anesthesiologists be available during TAVI for conversion preparation and intubation assistance in case of complications.

The limitations of this systematic review and meta-analysis are as follows. First, the few studies included in the analysis were retrospective or prospective reports lacking randomization, which may cause uncertainty in the results. Second, the lack of individual patient-level data prevented us from performing all analyses in prespecified patient subgroups, such as age, procedure time, and other characteristics that may yield additional clinical insights. There have been high-level comprehensive studies showing that anesthesia strategy has no significant impact on pneumonia, surgery conversion, and vascular complications. Meanwhile, for the prolonged intubation time and crossover percentages, insufficient numbers of reported original studies resulted in pooled results without statistical significance, which remains to be clarified by large-scale, high-quality randomized controlled clinical trials in the future. Although few studies have reported on LA to GA conversion, this subgroup of patients was included in the overall analysis of the LA cohort as an initial “intention-to-treat” strategy and therefore may impact the results. In addition, the abbreviations of variables included were not well-defined or accurately described in some studies, with no guidelines or consensus yet available. However, based on the strict inclusion and exclusion criteria of our research, we excluded potential risk factors as much as possible, which greatly increased the reliability of the study findings. Therefore, our systematic review and meta-analysis deserve careful interpretation, and the results may provide clues and reference for the design of relevant larger-sample and high-quality randomized controlled studies in the future.

## 5. Conclusions

Currently, no evidence exists to suggest which anesthetic strategy is superior during TAVI. Based on the results of this study, we found that LA (±CS) could improve productivity by reducing procedure time, fluoroscopy time, and hospital stay, with lower rates of cardiovascular drug use and risk of major bleeding, but may cause renal injury. Hence, we believe that both LA and GA can be used as anesthetic strategies to be implemented for patients with severe AS undergoing TAVI with high or medium risk. GA will be more convenient, safer, and surgeon-friendly when fusion imaging with transesophageal echo and fluoroscopy becomes standard. Except for the above, LA (±CS) may be a better option. The severity of the patient’s condition, their expectations, and the clinical experience of the surgical team all contribute to the choice of the appropriate anesthetic strategy. Future randomized controlled trials with higher quality and larger sample sizes will be necessary to confirm our findings and to determine which anesthesia strategy will be most beneficial for AS patients.

## Figures and Tables

**Table 1:** Baseline characteristics of included studies for meta-analysis.

Reference	Country	Group	N	Mean Age (year)	BMI (Kg/m^2^)	LVEF (%)
Brecker 2016	United Kingdom	LA	245	81.3 ± 6.2	NA	51.6 ± 13.8
		GA	245	81.6 ± 6.5	NA	52.7 ± 12.6
Gianni 2014	United Kingdom	LA	1095	82.5 ± 7.0	26.2 ± 4.9	53.5 ± 14.3
		GA	1712	81.4 ± 7.1	26.5 ± 5.0	53.4 ± 13.5
Attizzani 2018	American	LA	1988	81.9 ± 7.6	NA	NA
		GA	1988	81.7 ± 7.7	NA	NA
Benedicte 2011	France	LA	34	82.1 ± 10.1	NA	52 ± 15.5
		GA	91	81.9 ± 5.3	NA	50 ± 15.1
Sedat 2018	Turkey	LA	26	75.3 ± 6.9	26.7 ± 4.7	NA
		GA	23	77.7 ± 8.3	27.1 ± 3.9	NA
Mansoor 2019	American	LA	194	80 ± 8.8	NA	NA
		GA	224	81.8 ± 8.1	NA	NA
Holger 2020	Germany	LA	218	81.8 ± 5.3	26.7 ± 3.9	NA
		GA	220	81.4 ± 5.7	26.9 ± 3.8	NA
Motloch 2011	Austria	LA	41	82.6 ± 1.2	NA	53.6 ± 2.6
		GA	33	83.4 ± 0.6	NA	54.8 ± 2.3
Cedrick 2018	France	LA	168	81.8 ± 8.4	27.1 ± 5.8	52.3 ± 13.5
		GA	66	80.2 ± 7.5	26 ± 5.8	52 ± 14.8
Martins 2019	Portugal	LA	47	81 ± 6.1	NA	NA
		GA	102	81.6 ± 5.3	NA	NA
Errigo 2016	Italy	LA	310	82.7 ± 5.8	26.4 ± 5.5	NA
		GA	310	82 ± 5.4	26.4 ± 5	NA
Covello 2010	Italy	LA	42	79.5 ± 6.9	NA	NA
		GA	27	77.6 ± 7.9	NA	NA
Debry 2016	France	LA	52	81 ± 7.5	25 ± 4.5	51.6 ± 14.7
		GA	122	80.3 ± 8	27.1 ± 7.4	54.5 ± 13.2
Caroline 2015	Belgium	LA	66	85.6 ± 3.8	24.9 ± 3.8	NA
		GA	51	85.3 ± 4.6	25.4 ± 5.3	NA
Eskandari 2017	United Kingdom	LA	153	82.6 ± 6.7	27.5 ± 5.4	NA
		GA	153	82.4 ± 6.5	26.7 ± 5.6	NA
Soh 2017	Japan	LA	118	84 ± 6	21.5 ± 4.2	NA
		GA	118	83.5 ± 6	21.2 ± 4	NA
Babaliaros 2014	American	LA	70	82 ± 8	27 ± 5	49 ± 15
		GA	72	83 ± 8	28 ± 6	49 ± 15
Balanika 2014	Greece	LA	41	82 ± 6	NA	45 ± 15
		GA	57	81 ± 4	NA	45 ± 15
Bergmann 2011	Germany	LA	100	80.2 ± 6.6	26.9 ± 4.5	NA
		GA	51	81.1 ± 6.1	26.6 ± 4.9	NA
Goren 2015	Israel	LA	129	83 ± 5.4	27 ± 4	56 ± 8
		GA	75	83 ± 5.5	28 ± 5	57 ± 7
Palermo 2016	American	LA	44	85.4 ± 9.1	28.5	56.5 ± 12.3
		GA	21	79.6 ± 10.9	26.3	54.0 ± 14.3
Petronio 2015	Italy	LA	255	81.7 ± 6.8	NA	50.7 ± 12.2
		GA	255	81.4 ± 6.4	NA	51.1 ± 13.1
Mayr 2016	Germany	LA	31	82.9 ± 5.4	26.6 ± 3.9	52.7 ± 17.1
		GA	31	79.6 ± 7	26.1 ± 5.4	52.5 ± 13.2
Sandra 2014	Canada	LA	43	82.3 ± 6.2	NA	60.1 ± 10.3
		GA	101	81.8 ± 7.4	NA	52.2 ± 15.8
Elvin 2015	Turkey	LA	72	77.4 ± 8.7	27.2 ± 5.4	NA
		GA	79	76.3 ± 8.6	27.2 ± 5.1	NA
Wassim 2019	American	LA	154	82.1 ± 7.4	27.2 ± 5.3	54.3 ± 15.2
		GA	154	80.3 ± 9.3	29.9 ± 7.7	53.5 ± 17.2
Sarkis 2016	American	LA	467	82.9 ± 7.6	27.4 ± 6.8	52.8 ± 13.7
		GA	66	81.3 ± 10.6	31 ± 10.4	52.6 ± 14.5
Avais 2016	United Kingdom	LA	71	80.9 ± 6.8	27.6 ± 5.2	NA
		GA	145	80.2 ± 6.9	26.6 ± 5.6	NA
Wei 2017	China	LA	77	74.1 ± 6.4	23.3 ± 3.2	52.8 ± 13.6
		GA	36	76.0 ± 5.6	23 ± 3.2	51.4 ± 12.2
Oliver 2018	Germany	LA	2624	81 ± 6	27 ± 4.5	NA
		GA	2624	81 ± 5	27 ± 4.5	NA
Neumann 2020	Germany	LA	1027	81.9 ± 6.7	NA	NA
		GA	667	81.3 ± 6.6	NA	NA
Attizzani 2015	American	LA	116	81 ± 8	29.6 ± 8.7	51
		GA	91	81 ± 9	29.4 ± 7	49
Atsushi 2014	France	LA	403	83.3 ± 7.8	25.9 ± 4.6	53.4 ± 14.3
		GA	403	83.3 ± 6.9	25.9 ± 4.8	52.8 ± 13.7
Masanori 2013	France	LA	130	83.7 ± 7.1	25.7 ± 5	50.4 ± 14.1
		GA	44	84.7 ± 7	26 ± 4	45.1 ± 12.6

N, number; BMI, body mass index; LVEF, left ventricular ejection fraction; NA, not available. Values are mean, mean ± SD, or median (Q1, Q3).

**Table 2:** Baseline characteristics, medical conditions, and perioperative data of included studies for meta-analysis.

Reference	Age (Year)	CAD (%)	DM (%)	CRRT (%)	CVA (%)	COPD (%)	PVD (%)	Stroke (%)	NYHA III/IV (%)	EuroScore (%)
Brecker 2016	81.45 ± 6.4	54.2	24.3	13.3	12.5	NA	16.8	12.3	79	16.2
Gianni 2014	81.8 ± 7.1	20.1	25.3	7.6	11.9	25.3	NA	NA	78.4	20.4
Attizzani 2018	81.0 ± 8.3	65.2	35.3	3.3	12.2	43.7	25.7	12.2	79.3	NA
Benedicte 2011	82.6 ± 6.8	49.8	20.1	NA	62.7	25.9	7.3	11.3	82.6	23.9
Sedat 2018	76.4 ± 7.5	NA	NA	NA	NA	NA	NA	NA	4.1	26.6
Mansoor 2019	80.9 ± 8.5	NA	40.7	3.4	NA	23.9	NA	NA	85.6	NA
Holger 2020	81.6 ± 5.5	55.4	33.7	NA	NA	13.6	12.5	11.7	65.9	4.5
Motloch 2011	83 ± 0.9	43.2	28.4	NA	NA	10.8	NA	18.9	82.4	NA
Cedrick 2018	81.3 ± 8.2	46.1	23.9	NA	NA	30.8	28.6	7.7	57.3	22.6
Martins 2019	81.4 ± 5.6	40.9	28.9	NA	NA	18.1	26.2	14.1	64.4	NA
Errigo 2016	82.4 ± 5.6	25.2	29.2	1.3	NA	23.9	20	5.9	68.3	13.4
Covello 2010	78.8 ± 7.3	52	32	4	17	57	14	NA	67	26.5
Debry 2016	80.5 ± 7.9	52.2	31.6	22.9	NA	28.1	NA	13.7	83.9	20.1
Caroline 2015	85.5 ± 4.1	67.5	23.9	NA	20.5	31.6	NA	NA	65.8	26.3
Eskandari 2017	82.5 ± 6.6	38.6	23.5	1.3	14.1	30.8	10.2	14.1	76.8	NA
Soh 2017	83.8 ± 6	28	28	NA	NA	24.6	16.5	4.7	47.9	NA
Babaliaros 2014	82.5 ± 8	81	43.7	5.6	33.8	14.1	22.5	NA	88	NA
Balanika 2014	81.4 ± 4.8	NA	NA	NA	NA	NA	NA	NA	NA	27.4
Bergmann 2011	80.2 ± 6.5	59	NA	8	10	29	33	NA	NA	14
Goren 2015	83 ± 5.5	64	32	NA	9	15	26	3	96.6	NA
Palermo 2016	83.5 ± 9.7	66.1	33.8	1.6	13.9	27.7	4.6	NA	NA	13.4
Petronio 2015	81.6 ± 6.4	46.3	29.1	NA	NA	25.4	29.5	9.8	72.4	21.1
Mayr 2016	81.3 ± 6.2	NA	NA	NA	NA	NA	NA	NA	74.2	10.7
Atsushi 2014	83.3 ± 7.4	NA	25.1	NA	NA	25.8	12.9	10.5	76.7	18
Masanori 2013	83.9 ± 7.1	NA	22.4	NA	10.3	23.6	18.4	NA	65.5	23.8
Sandra 2014	82 ± 7	42.3	NA	NA	NA	9.7	NA	NA	84	NA
Elvin 2015	76.8 ± 8.6	60.3	18.5	NA	8.6	25.2	11.9	NA	NA	14.8
Wassim 2019	81.4 ± 8.3	NA	35	3	18	23	25	11	91	NA
Sarkis 2016	82.7 ± 8	73.7	34	NA	12.3	34.5	28.9	NA	87.8	NA
Avais 2016	80.4 ± 6.9	71.9	21.7	11.7	NA	37.8	14.7	NA	16	NA
Wei 2017	74.7 ± 6.2	46.9	17.7	15	NA	15.9	48.7	NA	74.3	NA
Oliver 2018	81 ± 6	54.1	13.8	2.7	2.9	12.6	14.4	NA	85.1	16
Neumann 2020	81.7 ± 6.7	48.8	28.7	NA	11.6	15.1	11.9	7.8	9	17.8
Attizzani 2015	81 ± 8.4	59.4	49.8	4.8	NA	38.6	23.2	19.3	84.1	NA

CAD, coronary artery disease; CRRT, continuous renal replacement therapy; CVA, cerebrovascular accident; COPD, chronic obstructive pulmonary disease; PVD, peripheral vascular disease; NYHA, New York Heart Association; NA, not available. Value are as mean, mean ± SD, or median (Q1, Q3).

## Data Availability

Data supporting the findings of this study can be found in the article or its supplementary material, and detailed data are available from the corresponding author upon reasonable request.

## Supplementary Materials

The following supporting information can be downloaded at: https://www.mdpi.com/article/10.3390/jcm12020508/s1, Figure S1: Risk of bias summary: review authors’ judgements about each risk of bias item for each included study; Table S1: Conversion rates and reasons for conversion from LA to GA; Table S2: Characteristics of patients with conversion from LA to GA.
