# Supplementary material for: Comparison of Safety and Effectiveness of Local or General Anesthesia after Transcatheter Aortic Valve Implantation: A Systematic Review and Meta-Analysis
Source: J Clin Med. 2023 Jan 7;12(2):508. doi: 10.3390/jcm12020508 (PMC9866516; doi:10.3390/jcm12020508)
Supplement: Supplementary file 1 [file jcm-12-00508-s001.zip › Funnel plot.pdf]

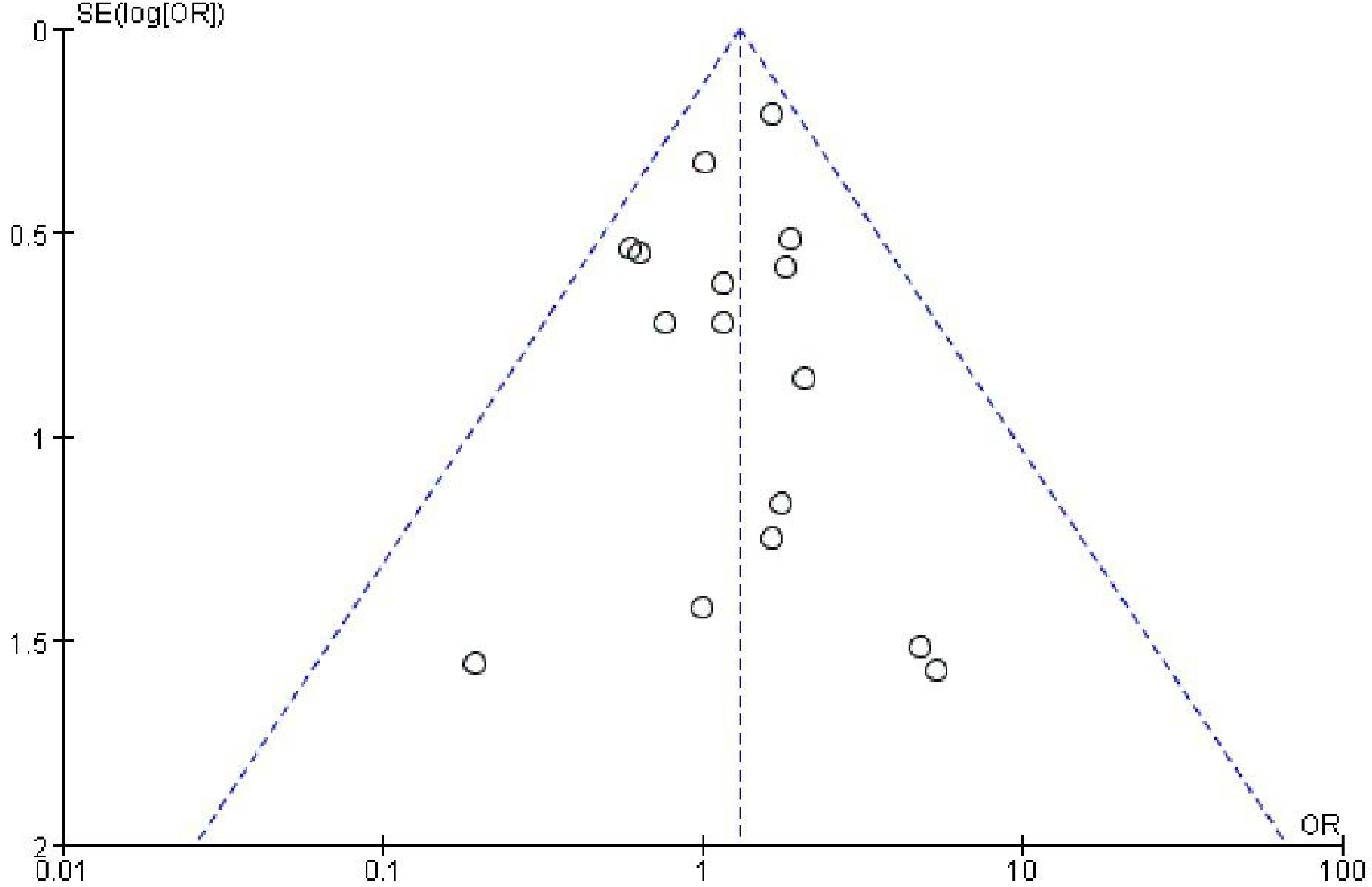

Funnel plot for incidence of AKI

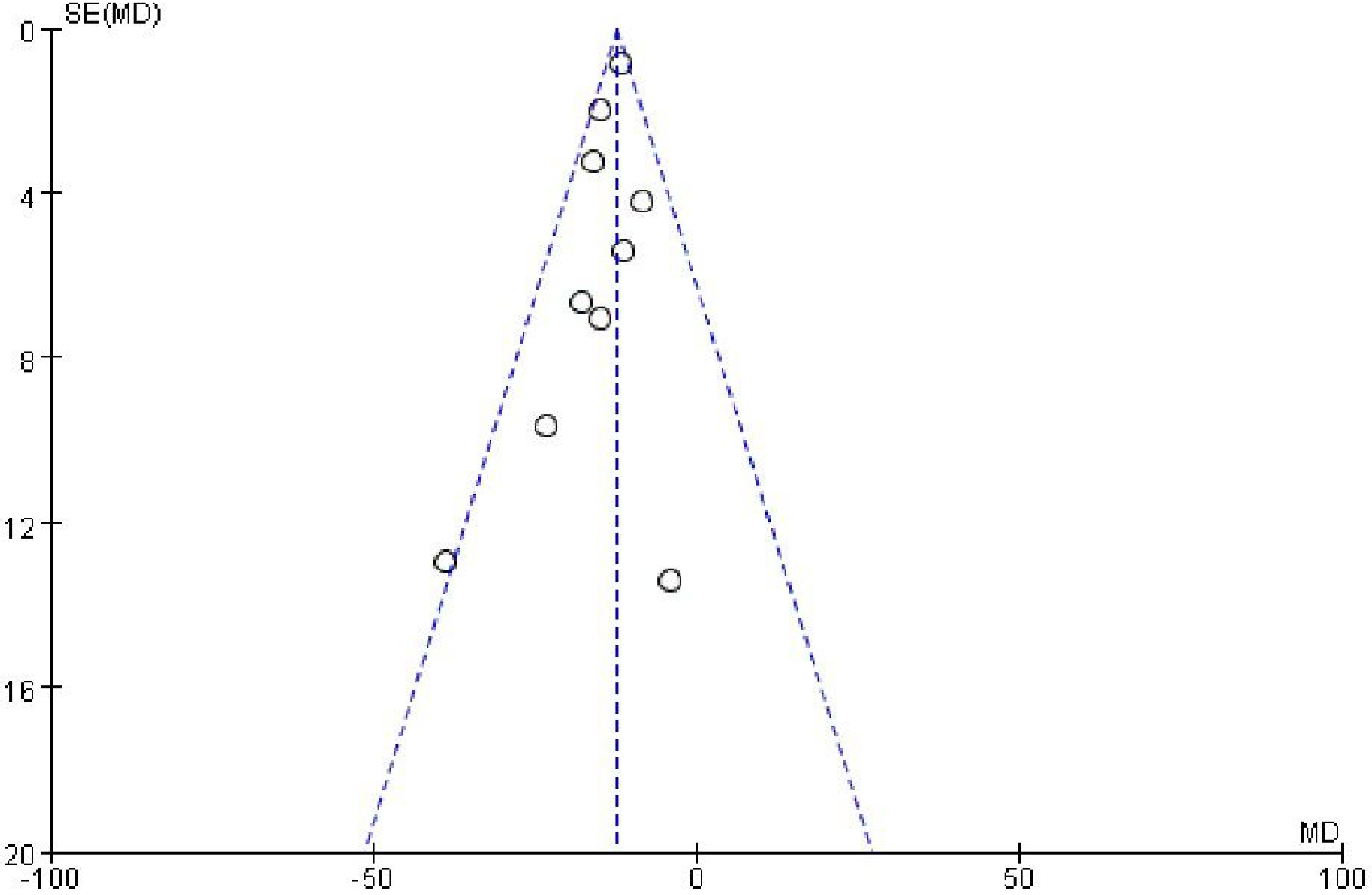

Funnel plot for procedural time

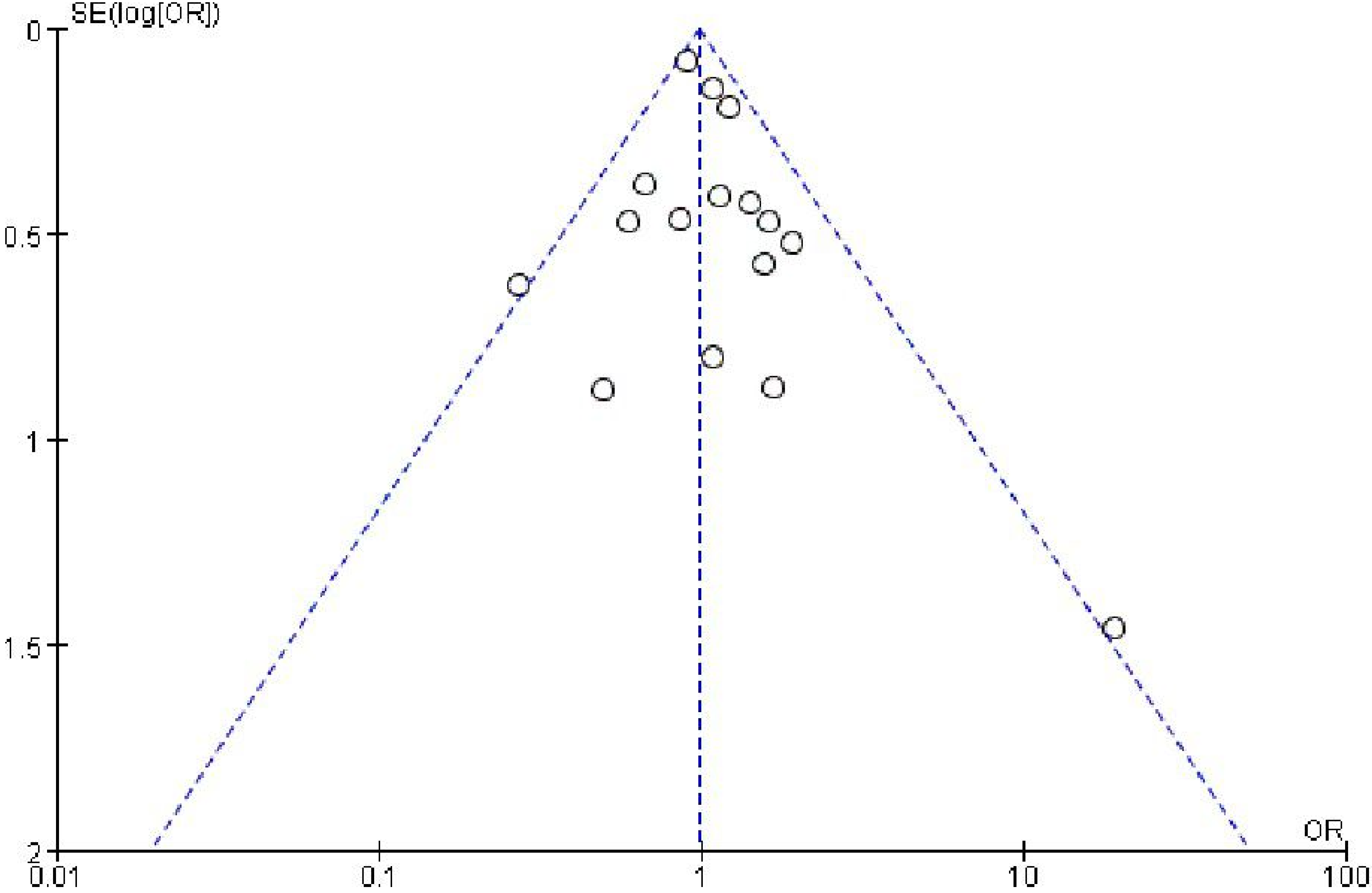

Funnel plot for the incidence of PPM implantation

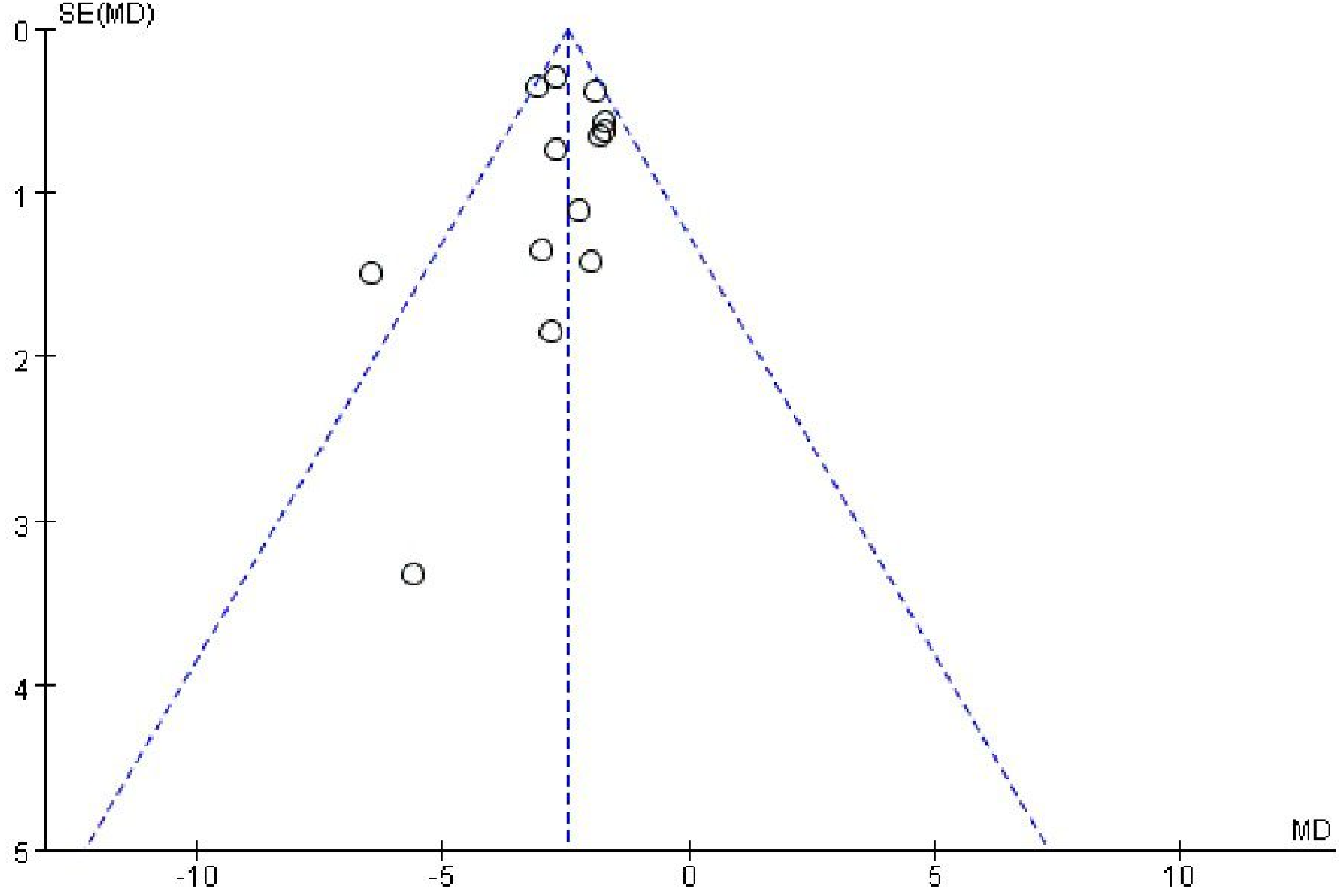

Funnel plot of length of stay

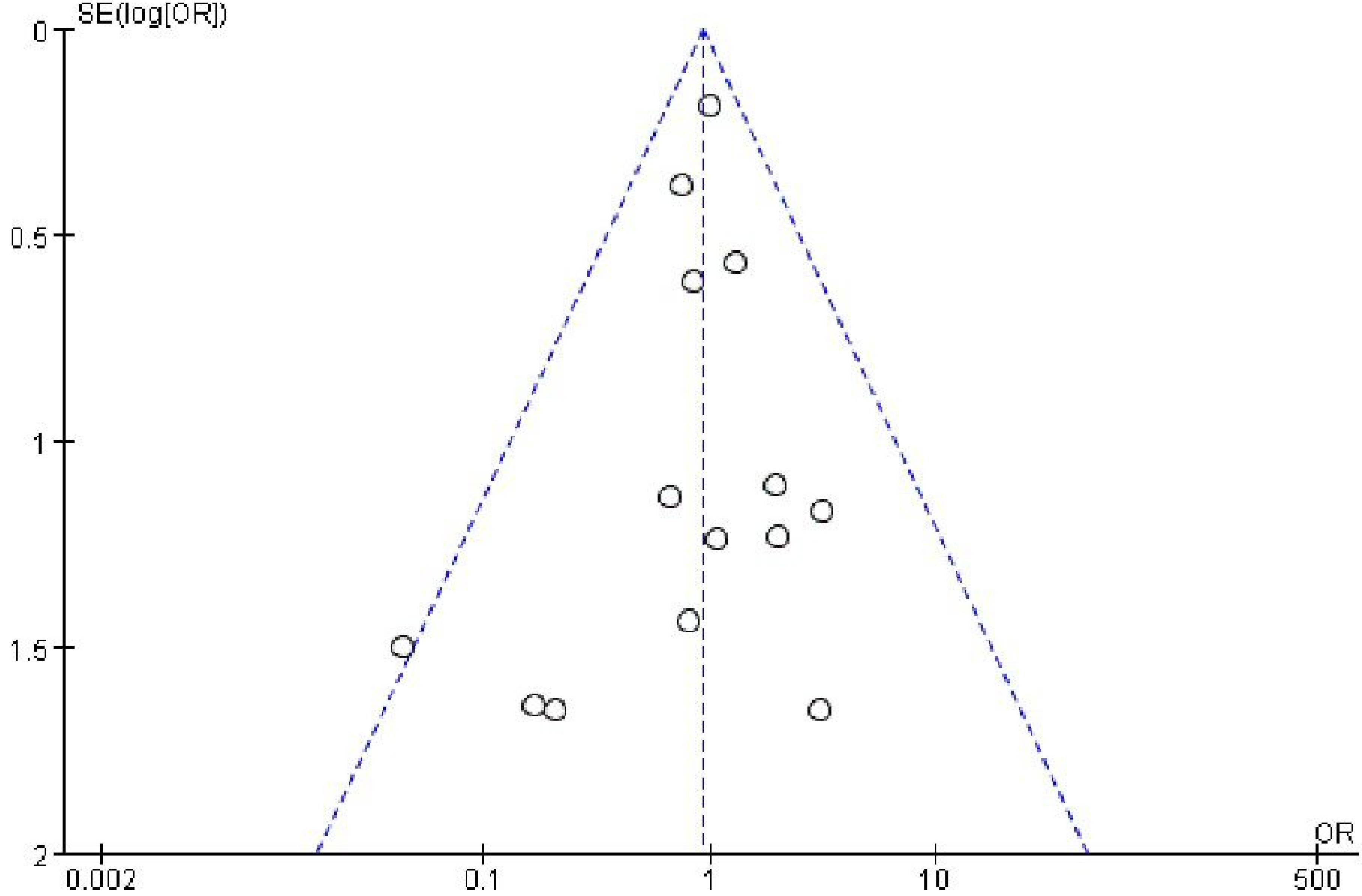

Funnel plot of the incidence of shock
