# Supplementary material for: Comparison of Safety and Effectiveness of Local or General Anesthesia after Transcatheter Aortic Valve Implantation: A Systematic Review and Meta-Analysis
Source: J Clin Med. 2023 Jan 7;12(2):508. doi: 10.3390/jcm12020508 (PMC9866516; doi:10.3390/jcm12020508)
Supplement: Supplementary file 1 [file jcm-12-00508-s001.zip › Publication bias.pdf]

Begg's Test

```
  adj. Kendall's Score (P-Q) =     -11
          Std. Dev. of Score =   18.27
           Number of Studies =      14
                          z  =   -0.60
                  Pr > |z|   =   0.547
                          z  =    0.55 (continuity corrected)
                  Pr > |z|   =   0.584 (continuity corrected)
```

Egger's test

Std_Eff	Coef.	Std. Err.	t	P>|t|	[95% Conf.	Interval]
slope	.0335035	.1894991	0.18	0.863	-.3793797	.4463866
bias	-.20946	.3452939	-0.61	0.555	-.9617906	.5428707

Publication bias of shock

Begg's Test

```
  adj. Kendall's Score (P-Q) =        12
          Std. Dev. of Score =     22.21
           Number of Studies =        16
                           z  =      0.54
                    Pr > |z| =     0.589
                           z  =      0.50 (continuity corrected)
                    Pr > |z| =     0.620 (continuity corrected)
```

Egger's test

Std_Eff	Coef.	Std. Err.	t	P>|t|	[95% Conf.	Interval]
slope	.3510862	.2079154	1.69	0.113	-.094848	.7970203
bias	-.1838042	.3885055	-0.47	0.643	-1.017066	.6494572

Publication bias of AKI

```
Begg's Test

  adj. Kendall's Score (P-Q) =     -24
          Std. Dev. of Score =   16.39
           Number of Studies =      13
                          z  =   -1.46
                    Pr > |z| =   0.143
                          z  =    1.40 (continuity corrected)
                    Pr > |z| =   0.161 (continuity corrected)
```

Egger's test

Std_Eff	Coef.	Std. Err.	t	P>|t|	[95% Conf.	Interval]
slope	-2.300829	.3971843	-5.79	0.000	-3.175026	-1.426632
bias	-.3513788	.6717797	-0.52	0.611	-1.829956	1.127198

Publication bias of length of stay

```
Begg's Test

  adj. Kendall's Score (P-Q) =        12
          Std. Dev. of Score =     22.21
           Number of Studies =        16
                          z  =      0.54
                    Pr > |z| =     0.589
                          z  =      0.50 (continuity corrected)
                    Pr > |z| =     0.620 (continuity corrected)
```

Egger's test

Std_Eff	Coef.	Std. Err.	t	P>|t|	[95% Conf. Interval]	
slope	-.0904396	.0972059	-0.93	0.368	-.2989255	.1180463
bias	.3869454	.4009197	0.97	0.351	-.4729419	1.246833

Publication bias of PPM implantation

```
Tests for Publication Bias

Begg's Test

  adj. Kendall's Score (P-Q) =       -7
          Std. Dev. of Score =    11.18
           Number of Studies =       10
                          z  =    -0.63
                    Pr > |z| =    0.531
                          z  =     0.54 (continuity corrected)
                    Pr > |z| =    0.592 (continuity corrected)

Egger's test
```

Std_Eff	Coef.	Std. Err.	t	P>|t|	[95% Conf.	Interval]
slope	-11.01294	1.046541	-10.52	0.000	-13.42627	-8.59961
bias	-.7581648	.4407885	-1.72	0.124	-1.774625	.2582954

Publication bias of procedural time
