# Supplementary material for: Comparison of Safety and Effectiveness of Local or General Anesthesia after Transcatheter Aortic Valve Implantation: A Systematic Review and Meta-Analysis
Source: J Clin Med. 2023 Jan 7;12(2):508. doi: 10.3390/jcm12020508 (PMC9866516; doi:10.3390/jcm12020508)
Supplement: Supplementary file 1 [file jcm-12-00508-s001.zip › Sensitivity analysis.pdf]

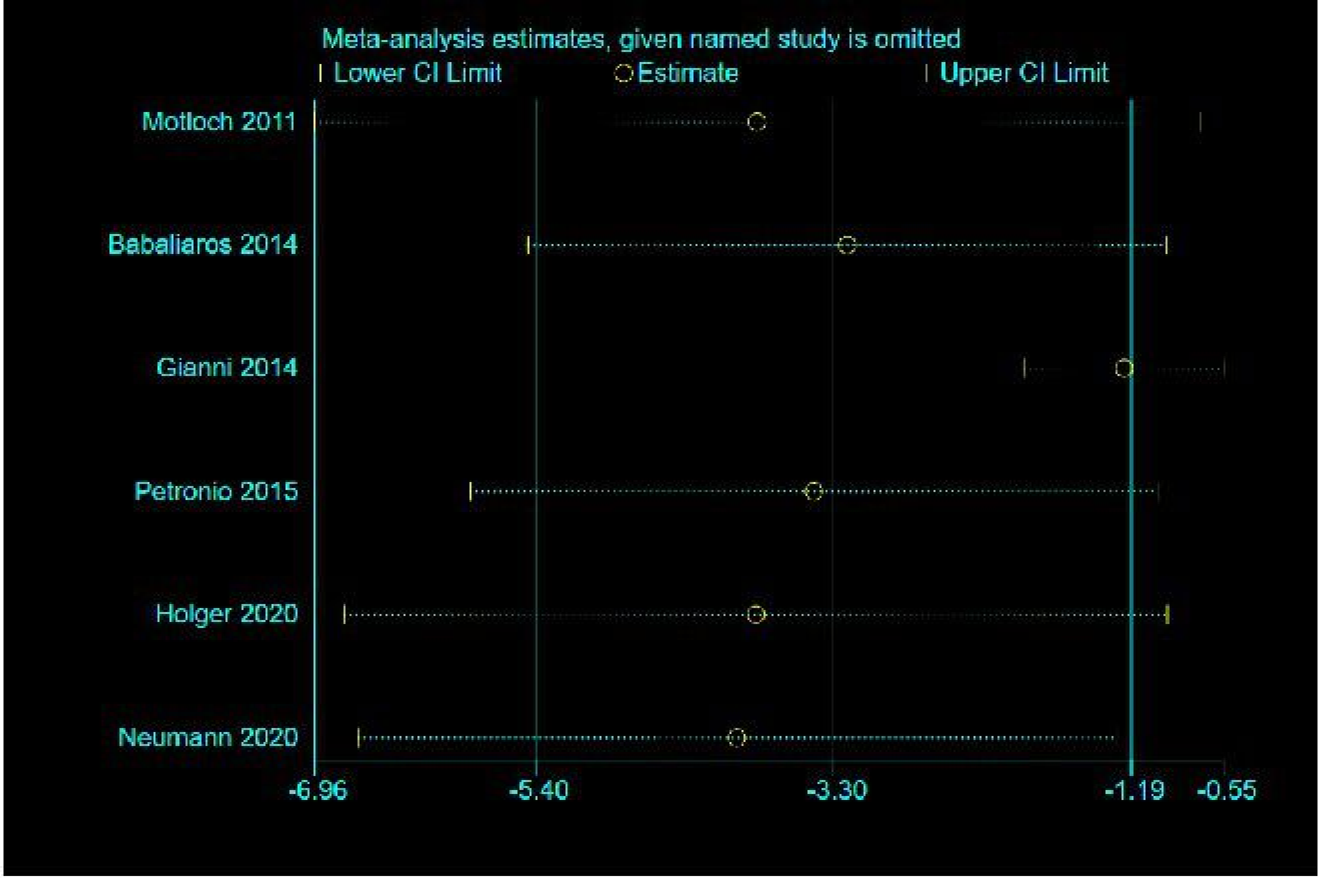

Sensitivity analysis for fluoroscopy time

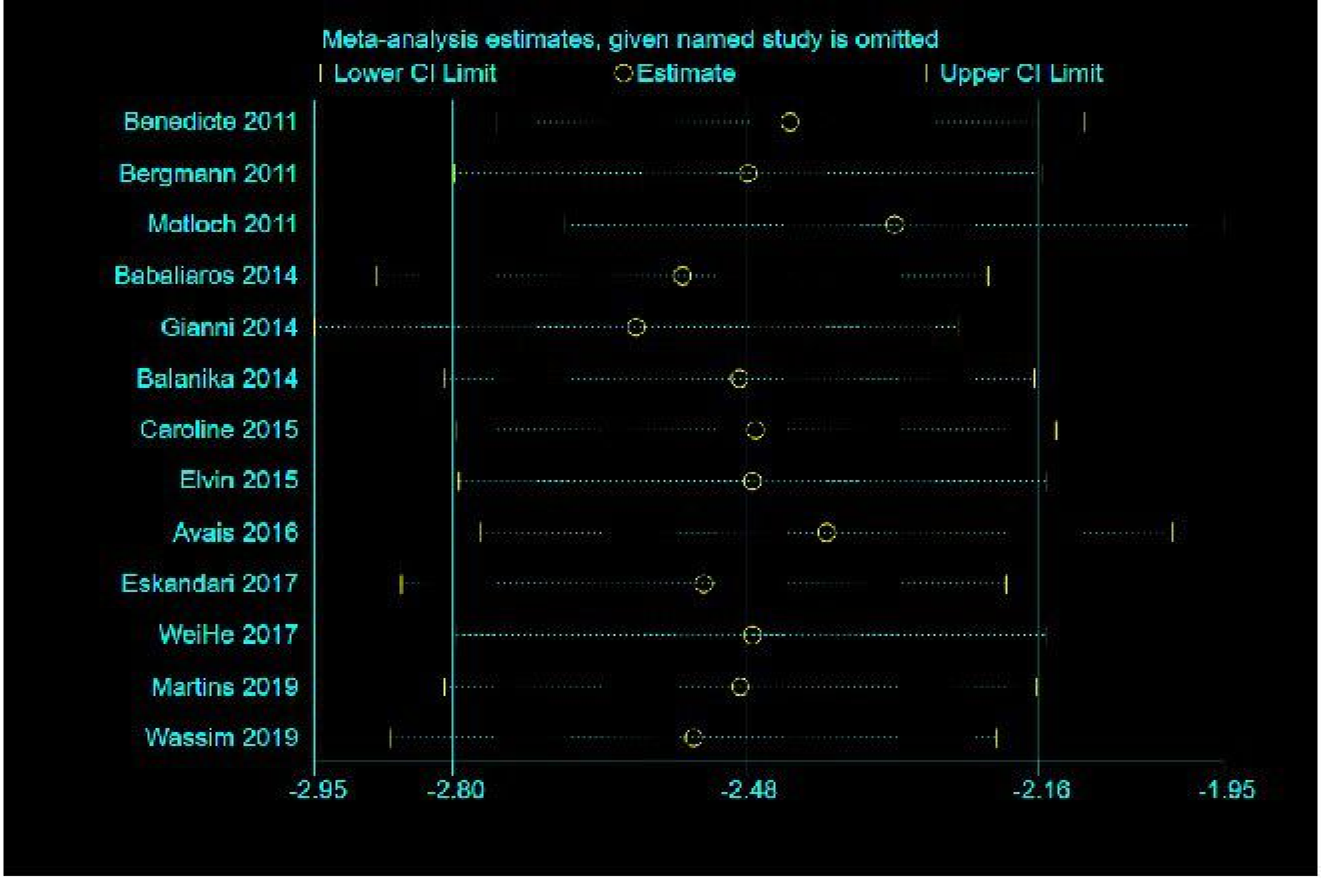

Sensitivity analysis for length of stay

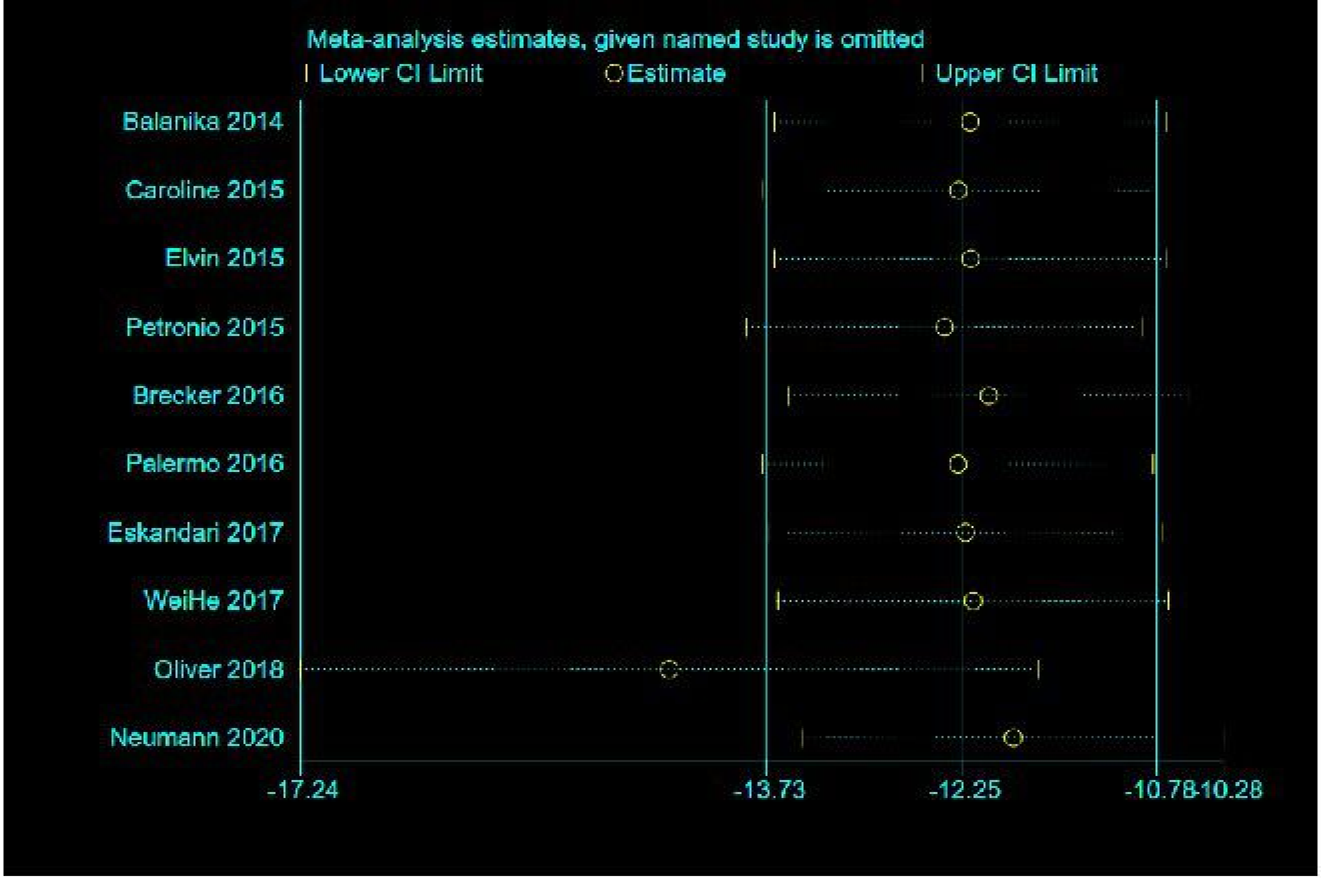

Sensitivity analysis for procedural time

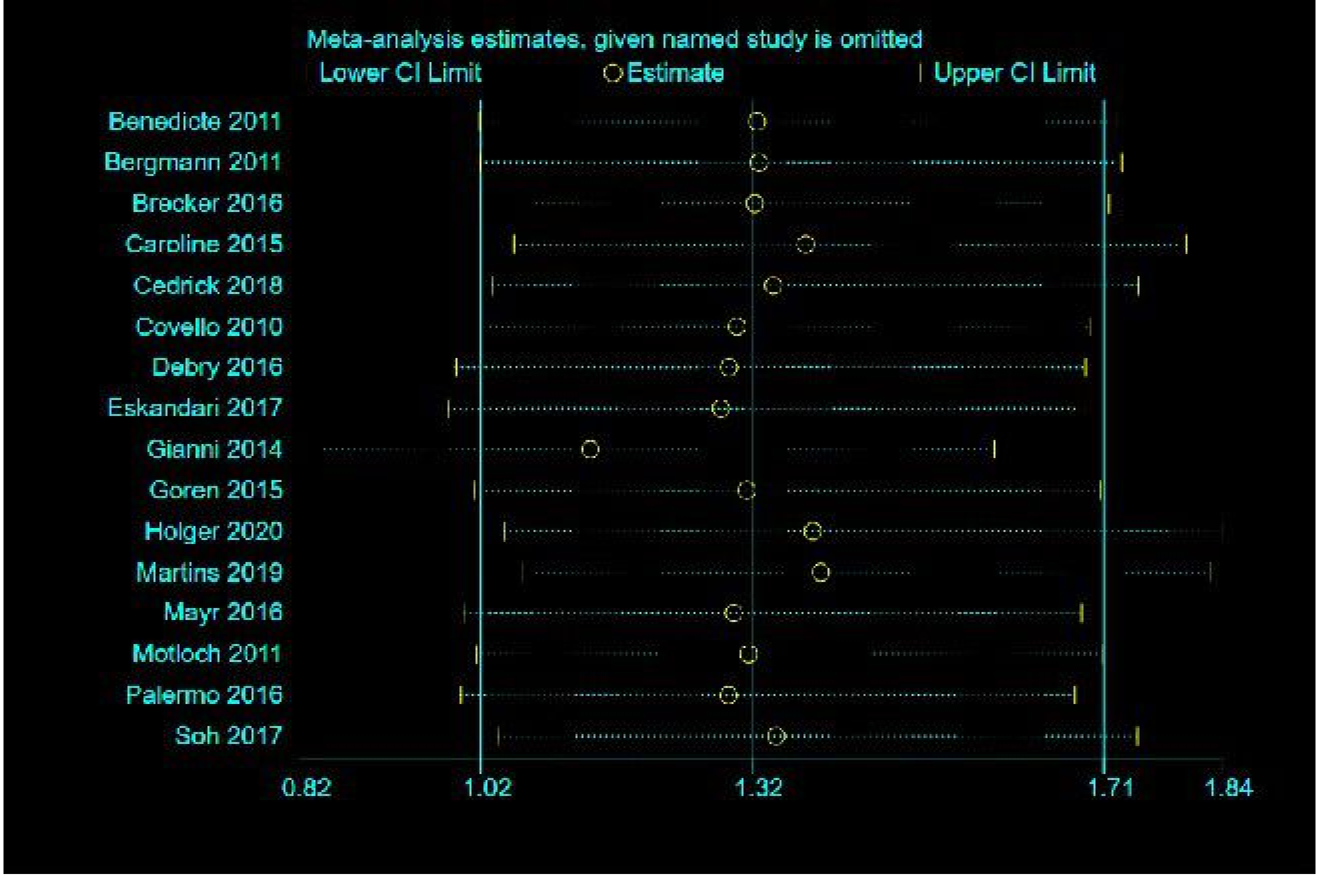

Sensitivity analysis for the incidence of AKI

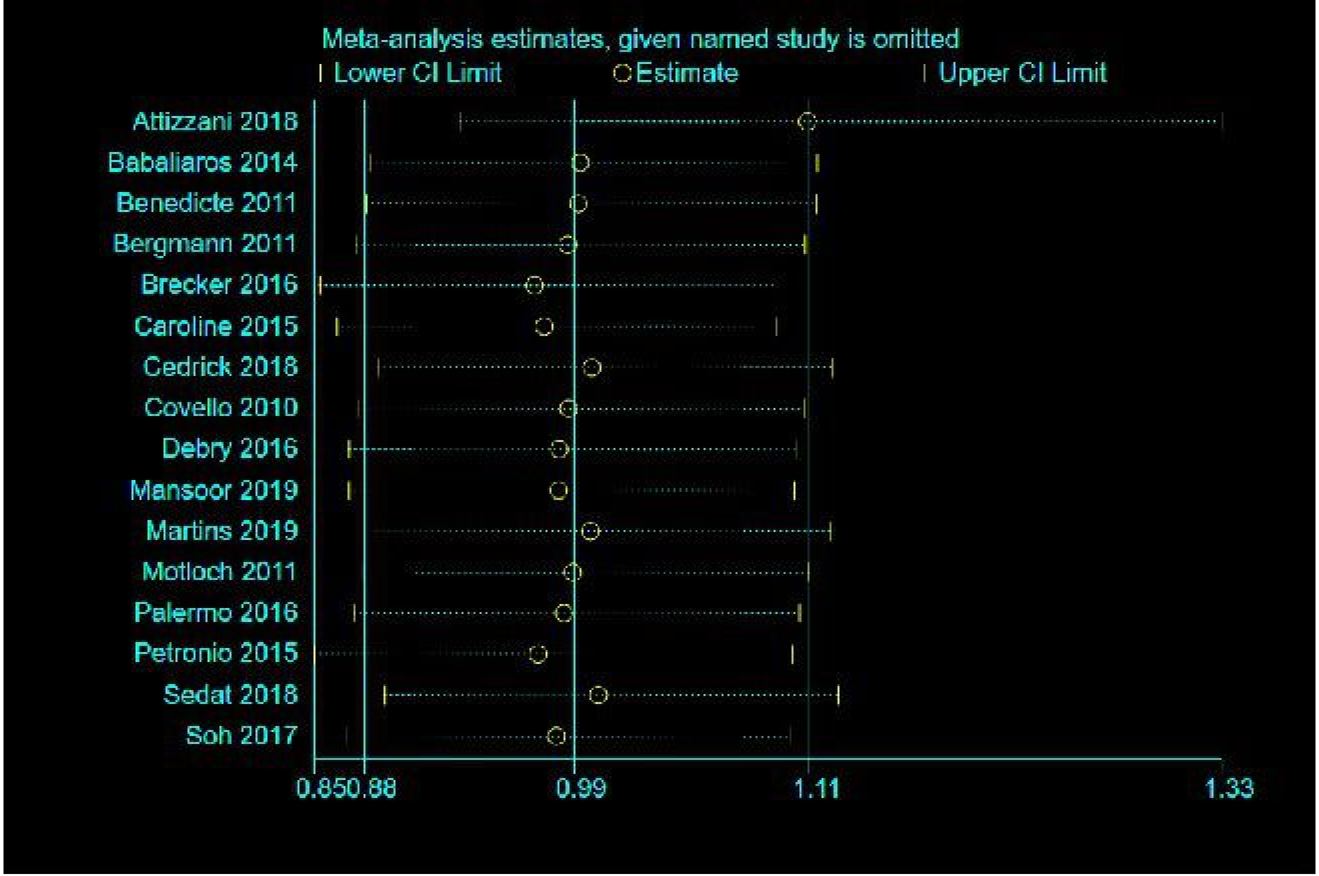

Sensitivity analysis for the incidence of PPM implantation

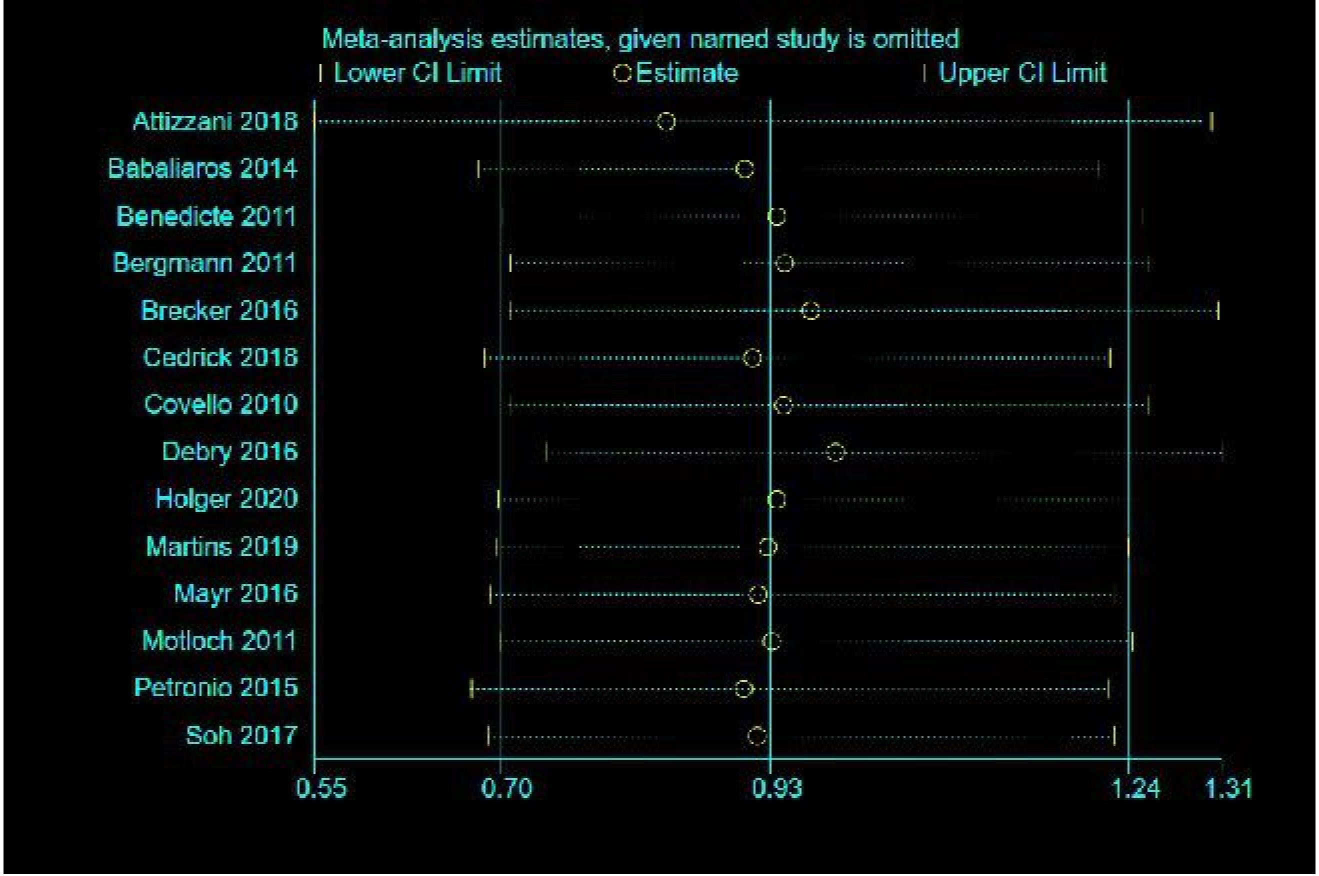

Sensitivity analysis for the incidence of shock
