# Supplementary material for: Comparison of Safety and Effectiveness of Local or General Anesthesia after Transcatheter Aortic Valve Implantation: A Systematic Review and Meta-Analysis
Source: J Clin Med. 2023 Jan 7;12(2):508. doi: 10.3390/jcm12020508 (PMC9866516; doi:10.3390/jcm12020508)
Supplement: Supplementary file 1 [file jcm-12-00508-s001.zip › Supplementary table 1.docx]

**Supplementary table S1.** Conversion rates and reasons for conversion from LA to GA.

| **Reference** | **Conversion Rate** | **Reasons (number of patients)** |
| --- | --- | --- |
| Attizzani 2015 | 3.4%  (4/116) | - pericardial tamponade (1) - emergent cardiac surgery due to valve embolization to the left ventricle (1) - restlessness of patient (1) - cardiac arrest (1) |
| Avais 2016 | 0%  (0/71) | - no conversions |
| Balanika 2014 | 0%  (0/41) | - no conversions |
| Behan 2008 | 11%  (1/9) | - uncooperative patient (1) |
| Bergmann 2011 | 17%  (17/100) | - vascular access complications (12) - pericardial tamponade (1) - uncooperative patient (2) - persistent ventricular fibrillation (2) |
| Brecker 2016 | 5.3%  (13/245) | - procedural complications, mostly related to valve positioning or vascular issues (9) - restlessness of patient (4) |
| Gauthier 2015 | 6.1%  (4/66) | - vascular complications requiring surgery (2) - uncooperative patient (2) |
| Goren 2015 | 4.7%  (6/129) | - hypotension (6) |
| Holger 2020 | 6.0% (13/218) | - restlessness of patient (3) - emergency surgery (5) - respiratory complications (3) - hemodynamic compromise such as arrhythmia, hypotension, cardiac arrest (2) |
| Kesimci 2016 | 16.7%  (12/72) | - restlessness of patient (11) - persistent ventricular fibrillation (1) |
| Kiramijyan 2016 | 12%  (56/467) | - hemodynamic compromise such as arrhythmia, hypotension, cardiac arrest (21) - conversion to surgical valve repair (9) - respiratory complications (9) - procedural complications such as left ventricular/annulus rupture, aortic dissection, vascular complications (9) - restlessness of patient (4) - laryngeal/glottic trauma due to TEE probe (4) |
| Mayr 2016 | 3.0%  (1/33) | - cardiopulmonary resuscitation, heart failure and respiratory distress (1) |
| Martins 2019 | 6.4%  (3/47) | - cefazolin hypersensitivity (1) - vascular complications with haemodynamic repercussion (2) |
| Neumann 2020 | 1.3%  (13/1027) | - pericardial tamponade (1) - persistent ventricular fibrillation (2) - procedural complications such as left ventricular/annulus rupture, aortic dissection, vascular complications (4) - dislocated valves need to be removed (1) - hemodynamic compromise such as arrhythmia, hypotension, cardiac arrest (2) - respiratory complications (1) - restlessness of patient (1) - failure of closure device (1) |
| Palermo 2016 | 2.3%  (1/44) | - restlessness of patient and hypotension (1) |
| Petronio 2016 | 2.9%  (27/917) | - procedural complications (NA) - uncooperative patient (NA) |
| Wassim 2019 | 1.2%  (5/425) | - no explanation (5) |
| Yamamoto 2013 | 4.6%  (6/130) | - pericardial tamponade (2) - cardiac arrest (2) - myocardial infarction (1) - stroke (1) |

**Overall conversion rate:** 182 out of 4157 patients, 4.4% (CI 3.8 to 5.1%)

NA: number not available
