# Supplementary material for: Comparison of Safety and Effectiveness of Local or General Anesthesia after Transcatheter Aortic Valve Implantation: A Systematic Review and Meta-Analysis
Source: J Clin Med. 2023 Jan 7;12(2):508. doi: 10.3390/jcm12020508 (PMC9866516; doi:10.3390/jcm12020508)
Supplement: Supplementary file 1 [file jcm-12-00508-s001.zip › Supplementary table 2.docx]

**Supplementary table S2.** Characteristics of patients with conversion from LA to GA.

| **Reason for conversion** | **Number of patients** | **Proportion (％)** |
| --- | --- | --- |
| Cardiac arrest | 3 | 1.6 |
| Pericardial tamponade | 5 | 2.7 |
| Hemodynamic compromise | 25 | 13.7 |
| Stroke | 1 | 0.5 |
| Persistent ventricular fibrillation | 5 | 2.7 |
| Myocardial infarction | 1 | 0.5 |
| Respiratory complications | 14 | 7.7 |
| Vascular access complications | 12 | 6.6 |
| Procedural complications | 22 | 12.1 |
| TEE-related trauma | 4 | 2.2 |
| Restlessness | 25 | 13.7 |
| Non-compliance | 5 | 2.7 |
| Other | 60 | 33 |
| Total | 182 | 100 |

Procedural complications include left ventricular/aneurysm rupture, aortic dissection, valve positioning failure, vascular complications, etc.

Other includes various incidental factors (equipment failure, allergies) or unexplained parts.
