# Supplementary figures and images for: Comparison of Safety and Effectiveness of Local or General Anesthesia after Transcatheter Aortic Valve Implantation: A Systematic Review and Meta-Analysis
Source: J Clin Med. 2023 Jan 7;12(2):508. doi: 10.3390/jcm12020508 (PMC9866516; doi:10.3390/jcm12020508)

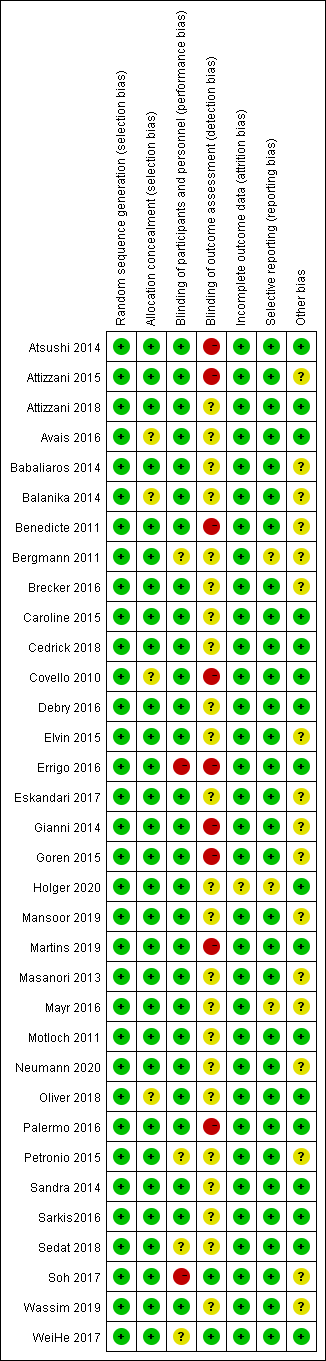

Supplement: Supplementary file 1 [file jcm-12-00508-s001.zip › Supplementary figure 1.png]
